# A neutron microscope using a nested Wolter-I condenser and a bank of diffractive–refractive achromatic objectives

**DOI:** 10.1107/S1600576726006254

**Published:** 2026-07-29

**Authors:** H. F. Poulsen, C. L. Andersen, N. Ravinet, J. Vila-Comamala, M. R. D. Veeraraj, E. B. Knudsen, P. K. Willendrup, S. Massahi, F. E. Christensen, D. D. M. Ferreira, M. Strobl, C. David, L. Theil Kuhn

**Affiliations:** a Department of Physics Technical University of Denmark 2800Kgs. Lyngby Denmark; b CHEXS ApS, Diplomvej 373B, 2800Kgs. Lyngby, Denmark; c PSI Center for Photon Science, Paul Scherrer Institute, Forschungsstrasse 111, 5232Villigen, Switzerland; d European Spallation Source ERIC – DMSC, 2800Kgs. Lyngby, Denmark; e Department of Space Technical University of Denmark Elektrovej 327 2800Kgs. Lyngby Denmark; f PSI Center for Neutron and Muon Sciences Paul Scherrer Institute Forschungsstrasse 111 5232Villigen Switzerland; g Department of Energy Conversion and Storage Technical University of Denmark Fysikvej 310 2800Kgs. Lyngby Denmark; Montanuniversität Leoben, Austria

**Keywords:** neutron imaging, neutron optics, multilayer optics, Fresnel zone plates, neutron microscopy, diffractive optics, refractive optics

## Abstract

We present a nested Wolter-I mirror design for a neutron condenser, which is based on established X-ray telescope technology. We demonstrate through simulations that it can increase the flux density at the ESS imaging instrument ODIN by up to two orders of magnitude. Experimental measurements of reflectivity and figure errors on a prototype mirror element confirm the technical feasibility of the approach. Then, we introduce design strategies for an imaging objective to fully exploit the condenser specifications while achieving spatial resolutions comparable to those of X-ray micro-computed tomography instruments. Analytically, we show that for monochromatic beams suitable solutions exist employing arrays of hundreds of identical objectives, realized either as compound refractive lenses (CRLs) or Fresnel zone plates (FZPs). To mitigate the inherent chromatic aberration of these optics, each individual objective could be replaced by an achromatic FZP/CRL combination. Key optical properties of the resulting microscope are estimated. This novel full-field microscopy concept for highly divergent, polychromatic neutron beams has the potential to improve temporal and spatial resolution for large samples and sample environments and to enable the simultaneous acquisition of hundreds of projections in neutron tomography.

## Abstract 2

A neutron microscope is presented comprising a Wolter-I nested mirror optic as condenser and a 2D array of neutron achromats as objective.

## 1. Introduction

Neutron imaging is a widely expanding field (Lehmann, 2015 ▸; Kardjilov *et al.*, 2018 ▸; Treimer, 2019 ▸; Strobl & Lehmann, 2024 ▸). The relatively low brilliance of neutron sources implies that imaging experiments typically require the use of a polychromatic beam with a relatively large divergence; in particular this is true for 3D and time-resolved studies. Historically, bright-field imaging studies have relied on placing a 2D detector downstream in close proximity to the sample. This pinhole camera approach has been exploited for a large variety of contrast mechanisms including attenuation contrast (Kallmann, 1948 ▸; Strobl *et al.*, 2009 ▸), phase contrast (Allman *et al.*, 2000 ▸; Paganin *et al.*, 2023 ▸; Østergaard *et al.*, 2023 ▸), dark-field imaging (Pfeiffer *et al.*, 2006 ▸; Strobl *et al.*, 2008 ▸; Bacak *et al.*, 2020 ▸; Shen *et al.*, 2025 ▸), diffraction contrast imaging (Santisteban *et al.*, 2001 ▸; Lehmann *et al.*, 2014 ▸), grain mapping (Peetermans *et al.*, 2014 ▸; Cereser *et al.*, 2017 ▸; Larsen *et al.*, 2025 ▸), and some versions of polarized neutron imaging for visualizing magnetic field distributions (Kardjilov *et al.*, 2008 ▸; Sales *et al.*, 2018 ▸; Sales *et al.*, 2019 ▸; Strobl *et al.*, 2019 ▸), magnetic domains (Manke *et al.*, 2010 ▸; Hiroi *et al.*, 2018 ▸; Jorba *et al.*, 2019 ▸) and electrical current distribution (Karimi *et al.*, 2025 ▸).

One basic limitation, however, is that the resolution deteriorates with larger distances between the sample and the detector, unless one compromises on the divergence of the incoming beam. This implies a trade-off between spatial resolution and time resolution, and, in general, limits the spatial resolution for extended objects and extended sample auxiliaries as well as for modalities such as polarized imaging where extended optics are required.

Inspired by imaging with light, X-rays and electrons, it is natural in such cases to consider a neutron microscope comprising a condenser optic upstream of the sample and an objective lens between the sample and the detector. The magnification intrinsic to most microscopes may also allow the use of more efficient detectors. Alas, such an instrument is yet to be realized.

In practice, several types of neutron optics have been demonstrated. Refractive optics, such as compound refractive lenses (CRLs), may provide high spatial resolution (Eskildsen *et al.*, 1998 ▸; Beguiristain *et al.*, 2002 ▸; Cremer *et al.*, 2005 ▸). However, the numerical aperture (NA) is fundamentally limited by the critical angle α = (2δ)^1/2^, where δ is the refractive index decrement. The resulting NA at 4 Å is at best 0.01 rad. Likewise, diffractive optical elements, such as Fresnel zone plates (FZPs), have been presented (Kearney *et al.*, 1980 ▸; Sacchetti *et al.*, 2004 ▸; Veeraraj *et al.*, 2025 ▸), but here the NA is limited by the manufacturing process to a few mrad. In contrast, the divergence accepted by the primary optics (in-pile neutron guide) is typically of the order of a few degrees (∼0.03 rad), implying that refractive and diffractive optics introduced downstream would only transmit a fraction of the incoming beam. Moreover, both types of optics are chromatic.

As an alternative, it has been suggested that nested concentric reflecting mirrors be used (Mildner & Gubarev, 2011 ▸; Khaykovich *et al.*, 2011 ▸; Liu *et al.*, 2013 ▸), in either a Wolter-I geometry (a paraboloid followed by a hyperboloid mirror) or a Wolter-II design (Abir *et al.*, 2020 ▸) (a convex paraboloid mirror followed by a concave hyperboloid mirror). These types of optics are well matched to the large divergence and size of the neutron beam and can be made achromatic using supermirrors. Notably Hussey *et al.* (2021 ▸) suggested using such optics as both condenser and objective. The potential increase in neutron flux is several orders of magnitude. However, the requirements for the shape errors of mirrors are of the order 1 mrad for a condenser with a focal length of 1 m, and a very demanding 1–10 µrad for an objective of focal length of 1 m. The work of Hussey *et al.* (2021 ▸) includes references to the state of the art.

In this paper we present progress towards realizing a neutron microscope. The presentation will focus on a high-flux reactor and spallation sources and more specifically on an optic for full-field neutron imaging at the European Spallation Source (ESS).

Initially, we present a design for a Wolter-I type condenser, that mimics the design of an existing X-ray telescope. The neutron condenser has a focal length of 1 m and comprises 28 shells with radii ranging from ∼10 to 50 mm. To reflect neutrons effectively over a broad wavelength range the individual depth-graded mirrors comprise multilayer coatings of Ni/Ti or NiC/Ti. We highlight the potential by showing full-scale ray tracing simulations using the *McStas* program (Willendrup & Lefmann, 2020 ▸; Willendrup & Lefmann, 2021 ▸) for a test at the upcoming ODIN beamline at ESS (Strobl, 2015 ▸). Despite the fact that this prototype condenser is not optimized for the beamline we find it may provide a flux density gain of 100 times in a focal spot size of 4 × 2 mm. Next, we present the results of feasibility tests for a single 60° mirror element of this design.

The resulting condensed beam can only be used for pinhole-type imaging of thin samples, as the divergence of the beam incident on the sample has increased from 0.5° to 3.0°, and consequently the depth of field has deteriorated. Moreover, performing tomography with such a beam implies that each projection is an average over a 3° rotation, which often is unacceptable. A spot size of 4 × 2 mm is also, in general, too big for scanning modalities. However, a highly intense 4 × 2 mm beam provides an adequate field of view (FOV) for microscopy, provided a suitable objective lens can be realized. As already mentioned, using a nested mirror design for the objective is two to three orders of magnitude more demanding in terms of shape errors. Instead, we propose a novel concept for the objective design based on combining diffractive and refractive optical elements. A spatial resolution of 2–10 µm is inherent to such optics; the challenge is how to make them compatible with the large divergence in the condensed beam and relevant for a broad energy band.

The solution suggested is a bank/array of objectives, each of them being a neutron achromat. In the second part of the paper, we review the state of the art of neutron CRL and FZP technology and achromat design with the aim of combining a converging FZP and a diverging CRL (Poulsen *et al.*, 2014 ▸). We describe the NA, FOV and bandwidth of banks of such achromats. Finally, we discuss the technical feasibility and data analysis for an instrument combining the condenser and the objective bank. While we do not detail the potential science case, one overriding ambition is to enable 3D neutron imaging with the same spatial and angular resolution as state-of-the-art X-ray micro-computed tomography (micro-CT), thereby addressing the needs of a large community in materials science.

## 2. Design and feasibility tests of Wolter-I type condenser

We have chosen a design that is an adaptation of the technology in the NuSTARtelescope probing 3–79 keV X-rays (Harrison *et al.*, 2013 ▸). The slope errors for such telescopes can be below 1 arcmin (Craig *et al.*, 2011 ▸), which is sufficient for a neutron condenser. The specific multilayer coating consists of a depth-graded multilayer following a power-law function (Christensen *et al.*, 2011 ▸).

### 2.1. Ray tracing

For ray tracing we have applied an idealized *m* = 3 supermirror model coating based on Ni and Ti layers. The selected coating model does not include figure error but includes a phenomenological model roughness for planar *m* = 3 mirrors, derived from Swiss Neutronics experimental data (Jacobsen *et al.*, 2013 ▸).

**Figure 1:**
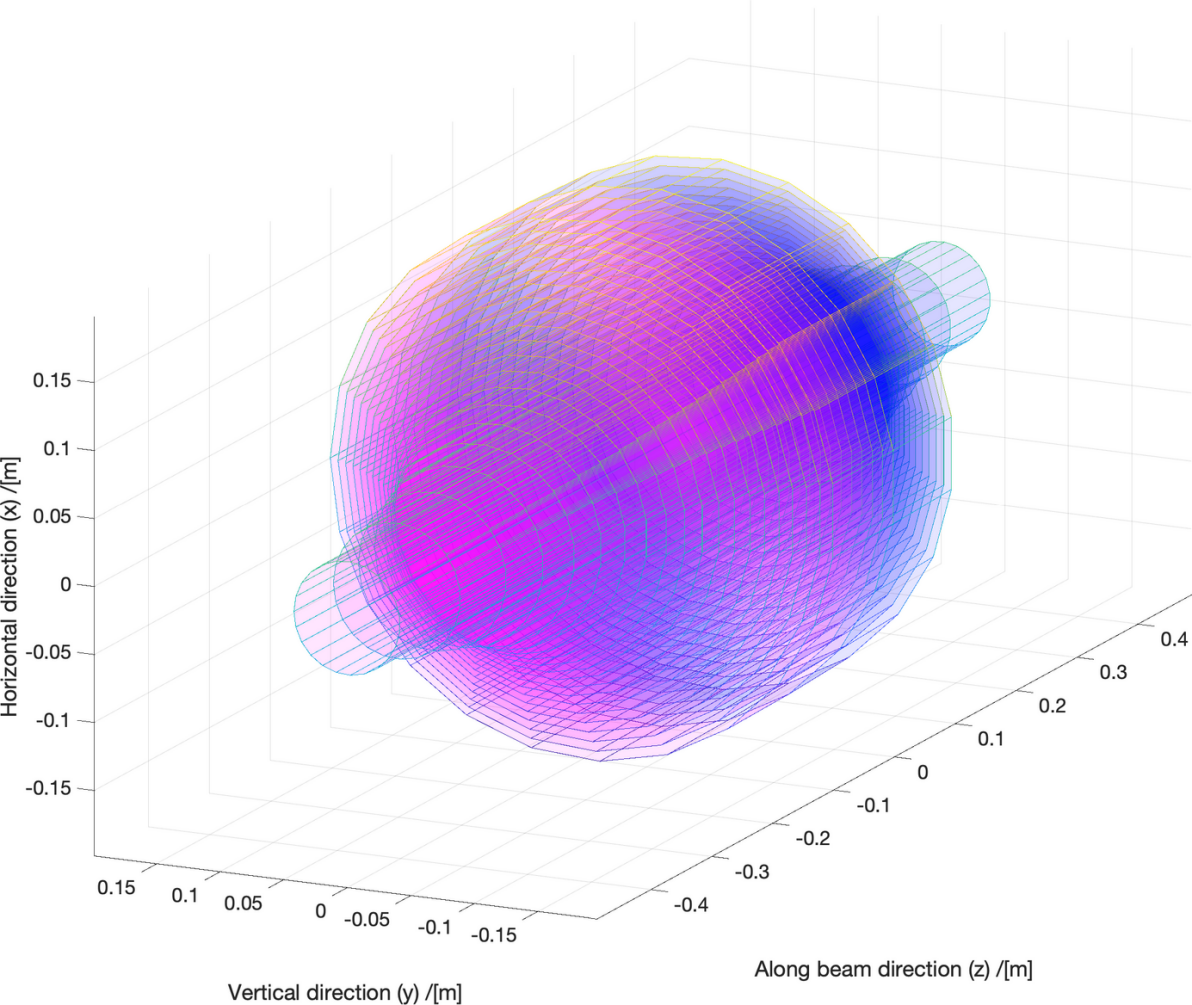
3D visualization of Wolter-I-optic-based condenser design. Lines have been added as a guide to the eye and the mounting-spokes visible in the simulation data have been omitted.

The design is outlined in Fig. 1 ▸. A total of 28 concentric conical layers are applied, all with a primary and secondary mirror in a Wolter-I geometry: the first is parabolic, the second hyperbolic. The layers are all confocal. The thickness is set to 0.2 mm, contributing a significant loss that is taken into account in the simulations. The radii vary from ∼10 to 50 mm. To achieve full coverage of this range, the mirrors have been designed with variable length, such that the inner mirrors are significantly longer than the outer ones. The nominal focal length of the optics, as measured from the center line of the optics, is 1 m. This set-up allows for 55 cm between the exit of the optics and the pivot point of the sample goniometer. For clarity of the geometrical parameters, all lengths and radii of the two conical shapes are listed in Appendix *A*[App appa] (Table 3).

The simulations were performed using *McStas*, specifically targeting a potential test of the optics at ODIN: an upcoming time-of-flight instrument with a peak wavelength at 3.7 Å (Andersen *et al.*, 2020 ▸). For our simulations, a model of the neutron beam transport from the design phase of ODIN (Strobl, 2015 ▸; Schmakat *et al.*, 2020 ▸) is used. The model includes a set of straight neutron guide sections with rectangular cross section and elliptical shape along the horizontal and vertical planes. The final elliptical section ends with a rectangular aperture of size 5 × 2.5 cm (W × H). The *McStas* implementation follows the concepts developed in the AstroX project (Knudsen *et al.*, 2018 ▸), where the mirrors are considered to have fully opaque back walls, *i.e.* there is no cross talk between the mirrors.

**Figure 2:**
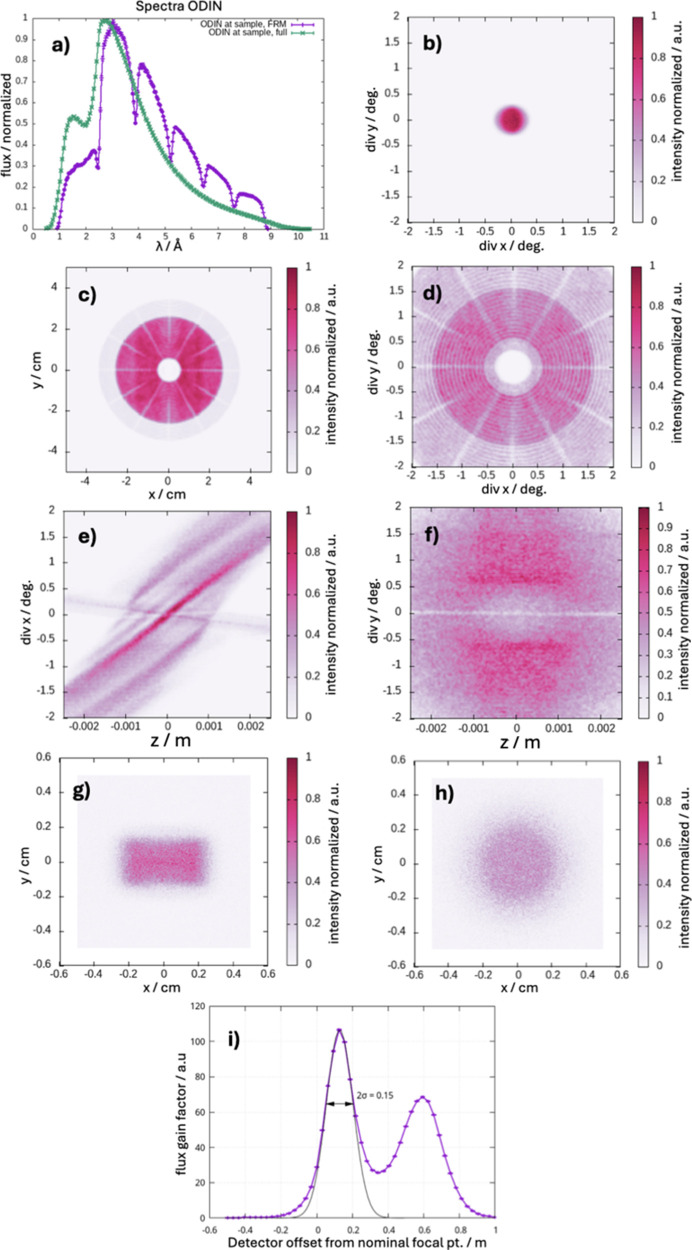
Results of *McStas* simulations of the Wolter-I type condenser for use at the ODIN instrument. (*a*) Spectrum of ODIN with (purple) and without (green) the frame-rate multiplication (FRM) chopper activated. (*b*) The divergence of the beam just upstream of the condenser. (*c*) Spatial distribution of the beam just downstream of the condenser. (*d*) 2D divergence distribution at the focal point. (*e*) Horizontal and (*f*) vertical divergence as a function of the position along the beam, *z*. (*g*) Spatial distribution, measured at the focal point. (*i*) Gain factor of neutron flux as a function of the distance from the nominal focal point. The first peak in (*i*) corresponds to the focal point; the second peak stems from a geometrical artifact caused by a single reflection on the Wolter-I optic. (*h*) The spatial distribution of the artificial beam spot at 0.6 m is correspondingly diffuse.

Fig. 2 ▸(*a*) shows the wavelength spectrum at the exit pinhole of the neutron guide. Gaussian fits to the divergence distribution at this pinhole result in widths (FWHM) of 1.03° and 1.06° in the horizontal and vertical directions, respectively. For the existing design of ODIN, the best position of the optics will be 10 m downstream of the pinhole. The dimensions of our Wolter-I optics design accommodate this. Fig. 2 ▸(*b*) shows the divergence of the beam at the *entry* of the condenser. We determine the angular spread of this beam to be 0.50°. In Fig. 2 ▸(*c*) the intensity distribution just after the condenser is shown; the spokes keeping the layers apart are clearly visible, as well as the central ‘20 mm hole’ which is not mirrored (this may be either blocked or used for a low-divergent modality in parallel).

Fig. 2 ▸(*d*) shows the angular distribution at the focal point. This is nearly uniform (except for the central hole and the spokes) with an FWHM of ∼3.0° (50 mrad). In the actual focal spot at a distance of 1.15 m from the center of the condenser [see Fig. 2 ▸(*g*)] the spot is rectangular with a size of ∼4 × 2 mm. For reference, Figs. 2 ▸(*e*) and 2 ▸(*f*) show the horizontal and vertical divergence, respectively, as function of the position along the beam. The strong anisotropy is caused by the design of the beam transport at ODIN. Finally in Fig. 2 ▸(*i*), the gain in neutron flux in relation to the unfocused case is shown as function of distance from the nominal focal point. The ‘focal spot’ is seen to be elongated over 15 cm along the beam and to exhibit a gain factor of 100 at the optimal distance. The secondary peak around 0.6 m is an artifact of stray neutrons, singly reflected through the optic. Fig. 2 ▸(*h*) shows the spatial extent of this artificial beam spot at 0.6 m. It may be removed by insertion of an aperture.

### 2.2. Feasibility tests

To assess the practical feasibility of this Wolter-I type condenser design, supermirror-coated, curved glass substrates were fabricated at the company CHEXS. We formed thin glass into 60° cylindrical segments with a curvature radius of 20 mm and a length of 100 mm. The glass is coated with a 256-bilayer NiC/Ti supermirror designed by CHEXS, utilizing DC magnetron sputtering. This coating targets an *m* value of 3, corresponding to a minimum bilayer thickness of 9.6 nm. For a complete shell in the Wolter-I optics design, six such segments are required.

**Figure 3:**
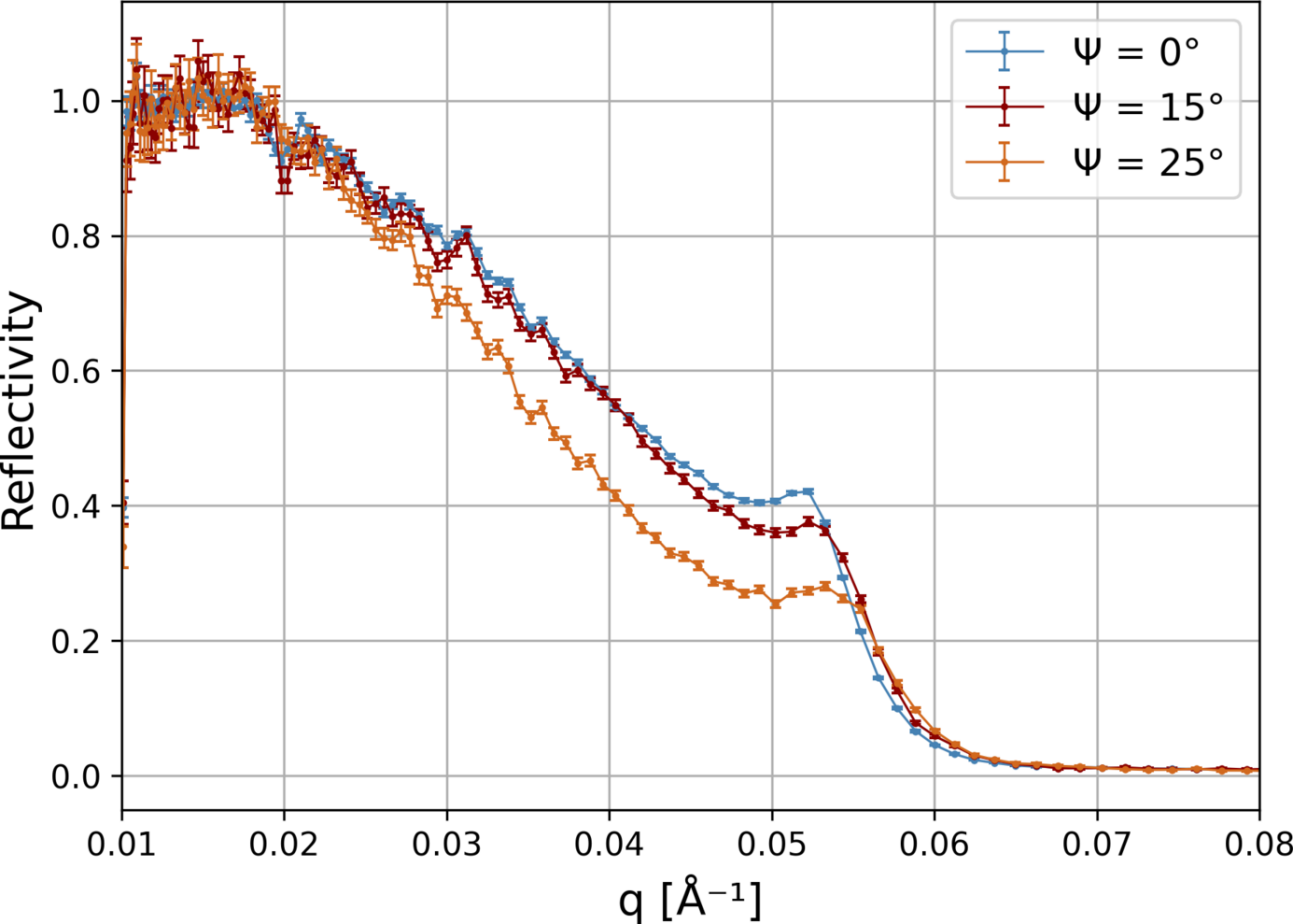
Measured neutron reflectometry of curved NiC/Ti supermirror-coated glass at three azimuthal positions, Ψ.

A supermirror segment was measured at the neutron reflectometer AMOR at PSI. Specular neutron reflectivity was recorded at three azimuthal positions (ψ = 0°, 15° and 25°), with ψ = 0° set at the center without distinction between the two symmetric sides of the arc. The resulting data are shown in Fig. 3 ▸. The measurements show that the mirror segment achieved an *m* value of 2.5, which corresponds to a minimum bilayer thickness of 11.2 nm. This coating parameter will be further optimized in subsequent experiments. The reflectivity at *m* = 2.5 was 0.30, 0.33 and 0.26, at Ψ = 0°, 15° and 25°, respectively.

An uncoated substrate was X-ray CT scanned, and the preliminary results indicate a figure error better than 1 arcmin. Additional characterization measurements are currently being performed on the coated substrates.

These results demonstrate that our first iteration of supermirror-coated curved glass substrates show promising performance and support further development towards a Wolter-I neutron condenser with performance comparable to that of the *McStas* simulations.

## 3. Objective bank solutions for a monochromatic beam

In this section we survey the possibilities for designing a bank (a 2D array) of either refractive or diffractive objectives subject to the following conditions.

 (i) A FOV of approximately 2 × 4 mm corresponding to the spot size provided by the condenser described in Section 2.

 (ii) An angular acceptance corresponding to the divergence of the incident beam, that is 3° (0.052 rad).

 (iii) A spatial resolution corresponding to state-of-the-art micro-CT machines: in the range 2–10 µm.

Another key design consideration is the focal length, which determines the available space for sample environments. In conjunction with the laboratory layout, particularly the sample to detector distance, it also determines the achievable magnification. Together with the detector characteristics, this in turn sets the attainable spatial and temporal resolution. With a view to existing neutron facilities, relevant design ranges are focal lengths of 30–150 cm and magnifications in the range of two to ten.

As both refractive and diffractive elements are chromatic we shall in this section assume a monochromatic beam. To facilitate the presentation, for both types of optics we initially summarize key expressions for a single optical element.

### 3.1. Geometry of a single-neutron CRL objective

The properties of a single-neutron CRL objective can be expressed analytically using geometrical optics. Following Simons *et al.* (2017 ▸), the CRL is assumed to comprise *N* identical lenslets, characterized by their radius of curvature at the apex, *R*, and the distance between the centers of neighboring lenslets, *T*. The focal length for each lenslet is then *f* = *R*/(2δ), while the physical aperture is 2*Y* = (*RT*)^1/2^. The focal length for the entire CRL (*f*_CRL_) is 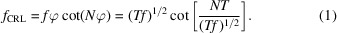
Here φ = (*T*/*f*)^1/2^ is introduced.

**Figure 4:**
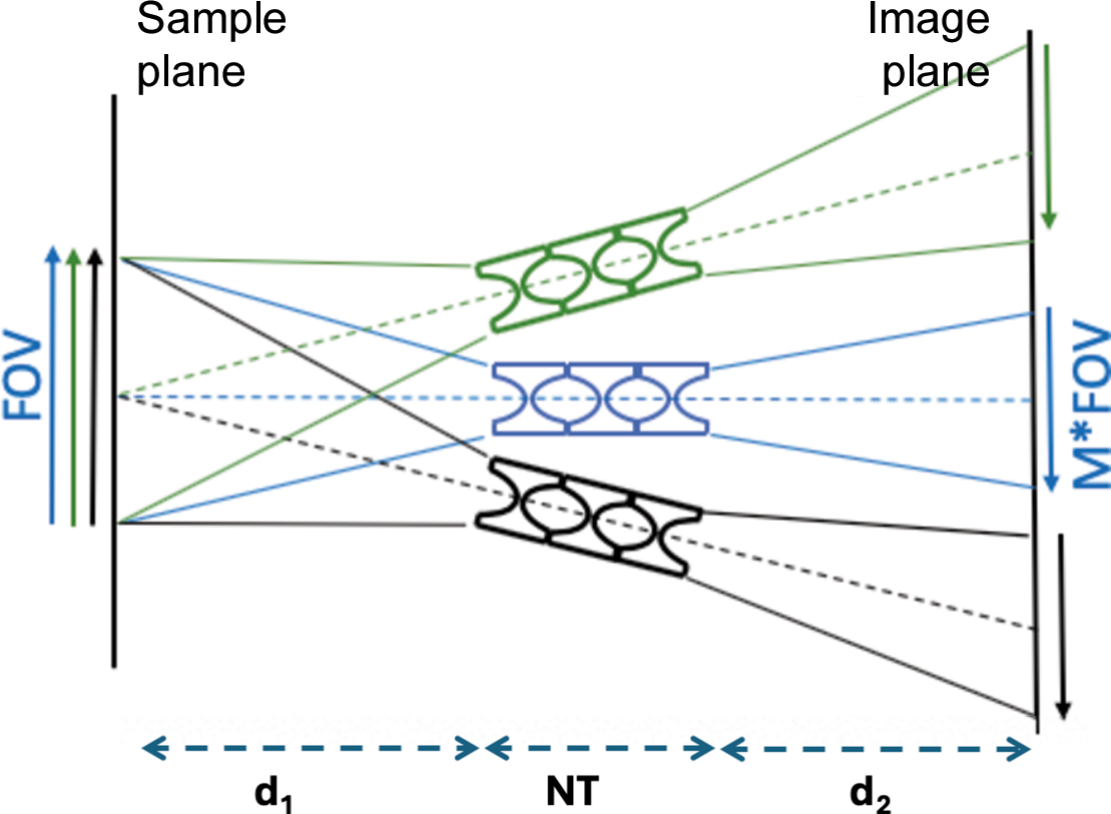
Illustration of the concept of a bank of objectives, where each objective is a compound refractive lens. The optical axes (dashed lines) coincide in the sample plane. Adapted from Leemreize *et al.* (2019 ▸).

Next, we consider an imaging system with *d*_1_ and *d*_2_ being the distances from the object plane to the entry of the CRL, and from the exit of the CRL to the image plane, respectively, as illustrated in Fig. 4 ▸. For the (unsigned) magnification we have 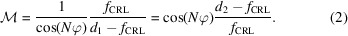
Closed expressions for NA and FOV are derived by Simons *et al.* (2017 ▸). Following conventions in that work, CRL numbers for FOV and NA numbers in this paper represent the FWHM. Leemreize *et al.* (2019 ▸) argued that a neutron CRL typically can be approximated by a transparent lens, where the attenuation of the neutrons within the parabolic part is neglected. For some optical properties, the CRL then simply acts as a collimator with dimensions given by the physical aperture 2*Y* and the length *NT*. The NA is in this case 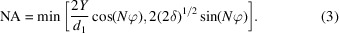
The first term reflects the limitation by the CRL as a collimator and the second term its limitation in terms of refractive power. The cosine factor in the first term originates in the fact that the neutron trajectory within the CRL approximately is a sinusoid with period 2π*N*φ. In the transparent lens case we have the following expression for the FOV:
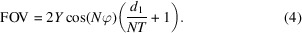
The maximum NA available is realized for *N*φ = π/4. Then equation (1) and equation (4) become 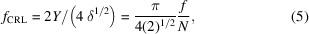

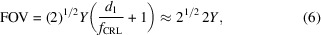
where the approximation is valid for large 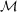.

The main technical limitation for CRLs is manufacturing precision. The technology required can be similar to that used for X-ray CRLs, where sub-micrometre resolution is standard (Schroer & Lengeler, 2005 ▸; Cederström *et al.*, 2005 ▸; Antipov *et al.*, 2016 ▸).

### 3.2. A CRL-based objective bank

Following Leemreize *et al.* (2019 ▸), we selected two materials as prime candidate materials for scale up: diamond, due to its superior specifications and prior use, and MgF_2_, which is easily available and can be machined by milling machines. Moreover, both are readily available as single crystals. This is important as both materials exhibit a pure absorption cross section that is two orders of magnitude smaller than the coherent scattering cross section.

In Table 1 ▸, we provide key parameters for these materials. The tabulated values are for cold neutrons, as these are relevant for most of the applications presented. Writing the refractive index *n* = 1 − δ, the decrement δ is positive for both materials, similar to the case for X-rays but an order of magnitude larger. We have δ ∝ λ^2^, where λ is the neutron wavelength.

It appears that the incident divergence provided by the Wolter-I condenser is about five times larger than the maximum NA for a C-based CRL objective, and about a factor of ten times larger in the case of an MgF_2_-based CRL objective. Central to this work is the proposal to overcome this hurdle by using a 2D bank of objectives, tilted such that the optical axes of the various CRLs coincide at the origin of the sample plane. This is illustrated in a cross section through the optics in Fig. 4 ▸. (The bank of objectives will comprise elements that are also out of the plane of view.)

There are four independent variables: *N*, *R*, *T* and *d*_1_, which determine the four optical properties *f*_CRL_, 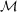, NA and *d*_2_. Reversely, specifying the optical properties and/or the layout of the laboratory (and thereby *d*_2_) defines a design of the CRL and the working distance *d*_1_. We now discuss optimizing these parameters to conform as closely as possible to the condenser specifications.

First we aim for the maximum achievable NA, tabulated in Table 1 ▸, as this will reduce the number of CRLs in the bank. Requiring FOV = 4 mm implies that 2*Y* = 4/2^1/2^ mm. From equation (5), it follows that the focal length *f*_CRL_ is 130 mm for diamond and 265 mm for MgF_2_. As an example for diamond this is achievable by the design (*N*, *R*, *T*) = (59, 1 mm, 2.8 mm), which is technically feasible. Notably, the maximum NA appears in the thick lens limit, here *NT* = 46 cm.

In terms of resources such a set-up would require approximately 25 (diamond) or 100 (MgF_2_) individual CRLs and an appropriate detector coverage. When relevant, the intensities in the individual images may be superposed to form one resulting 2D image of the 4 × 2 mm region of interest. For tomography, each column of the array provides one projection in the neutron tomogram.

Notably, a CRL objective bank has previously been implemented for X-ray laboratory sources. This was done by Opolka *et al.* (2021 ▸), who report on a multi-lens array full-field X-ray microscope manufactured in Si by means of lithography.

An additional constraint applies to ensure that the partial images provided by neighboring CRLs do not overlap on the detector. The condition for a complete spatial and angular sampling is 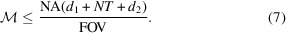
The optimal design of a CRL bank will depend on the minimum focal length acceptable (in order to comply with sample auxiliaries) and the maximum sample-to-detector distance *L* = *d*_1_ + *NT* + *d*_2_ (as this will determine the magnification). An example of nominal design parameters is *f*_CRL_ = 300 mm, FOV = 4 mm and *L* = 5 m. Coupled with NAs approaching those listed in Table 1 ▸, the inequality in equation (7) is fulfilled for magnifications of five or less.

In conclusion, for monochromatic beam operation, this objective bank is well suited for inclusion in a micro-CT neutron microscope based on the Wolter-I condenser. The available space around the sample – as defined by the focal length – adheres to the design specifications stated at the beginning of this section. It also matches the FOV, and the magnification enables the combination of a micrometre-sized spatial resolution with a magnification of 5 or more. Moreover, the depth of field = *y*_s_/NA (with *y*_s_ being the spatial resolution in the sample plane) will be hundreds of micrometres.

### 3.3. Geometry of a single FZP-based objective

An FZP is a diffractive optical element composed of concentric rings with radially decreasing line widths. In the following we consider a neutron phase FZP where the rings/zones alternate between being phase-shifting and non-phase-shifting (Kearney *et al.*, 1980 ▸; Sacchetti *et al.*, 2004 ▸; Altissimo *et al.*, 2004 ▸; Veeraraj *et al.*, 2025 ▸). Phase FZPs have a higher diffraction efficiency than amplitude FZPs and they also suppress the zeroth-order diffraction beam, making them better suited for imaging applications.

An ideal phase FZP has a maximum diffraction efficiency of 40.5%, which can be achieved by choosing a zone thickness Δ*T*_π_ that induces a π phase shift in the incident radiation, 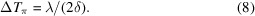
For thermal and cold neutrons, with wavelengths ranging from 1 to 10 Å, the Δ*T*_π_ required is typically of the order of micrometres to a few tens of micrometres. The diffraction efficiency can be significantly improved by using a blazed FZP, in which the zone profiles consist of steps to approximate a continuous phase ramp (Fabrizio *et al.*, 1999 ▸; Mohacsi *et al.*, 2016 ▸).

The optical properties of the FZP are governed by the outermost zone width, Δ*r*. For thermal or cold neutrons the focal length of a single FZP (*f*_FZP_) will be much larger than the diameter of the optic (*D*). In the paraxial approximation we have 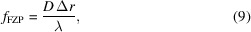

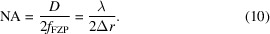
Because the FZP is a thin lens, the following relationships apply in an imaging set-up: 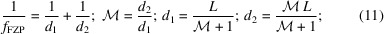
with *L* being the length of the imaging system (*L* = *d*_1_ + *d*_2_).

The FOV of an FZP is discussed by Howells *et al.* (2017 ▸). While aberrations exist for off-axis imaging, for *e.g.* 5 keV X-rays, these remain below 100 nm for objects with a size as large as 10× the zone plate radius. From this we assume that such distortions will not have a noticeable impact on the resolution in neutron microscopy.

**Figure 5:**
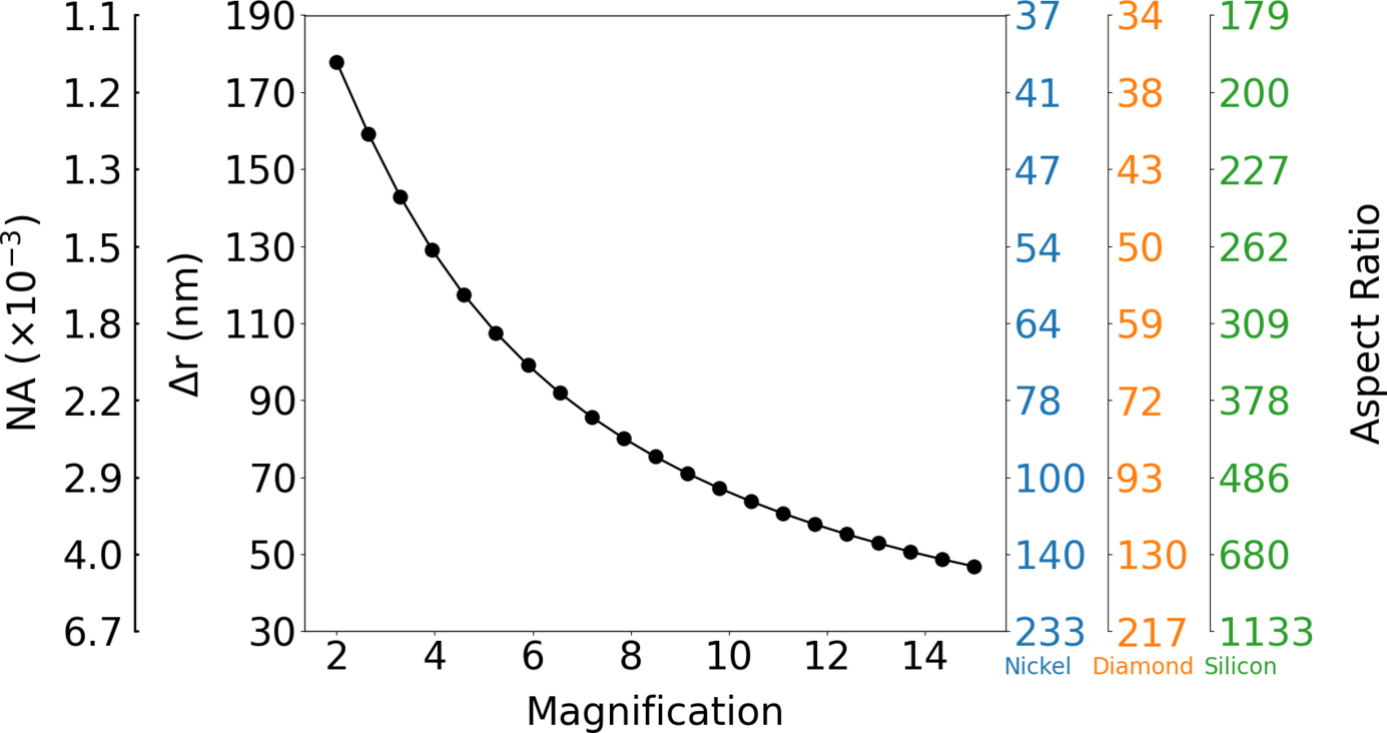
Manufacturing specifications for a single FZP objective in terms of outermost zone width Δ*r* as a function of magnification for three materials. The corresponding NA is shown. Moreover the required ARs of the outermost zone for three materials for maximum efficiency are shown. The example relates to a fixed diameter *D* = 4 mm, wavelength λ = 4 Å and an experimental laboratory allowing *L* = 8 m.

From equations (8), (9) and (10), the critical performance parameter of the FZP is the aspect ratio (AR) of the outermost zone, AR = Δ*T*_π_/Δ*r*. Table 2 ▸ provides a list of materials that can be considered for the fabrication of neutron FZPs and key optical parameters. We have δ ∝ λ. In Fig. 5 ▸, as an example, we show the manufacturing requirements with respect to AR for these three materials and for relevant settings of *D*, λ and *L*.

It can be seen from Fig. 5 ▸ that, to effectively match the incident divergence from the condenser, the FZP would require very high aspect ratio nanostructures, which are technically challenging to fabricate. Strategies to improve and develop these ARs are presented by Chang & Sakdinawat (2014 ▸), Mohacsi *et al.* (2016 ▸) and De Andrade *et al.* (2021 ▸). However, an FZP with Δ*r* around 40 nm roughly matches the NA_max_ of a diamond CRL, and would still have five times smaller NA than the incoming divergence from the condenser described in Section 2.

### 3.4. A bank of FZP objectives

In analogy to the CRL case discussed above, we analyze how to mitigate the mismatch between the NA of the FZP and the incident divergence of the condensed beam by employing a bank of FZPs. The considerations in relation to avoiding overlap of partial images, *cf*. equation (7), apply here as well.

**Figure 6:**
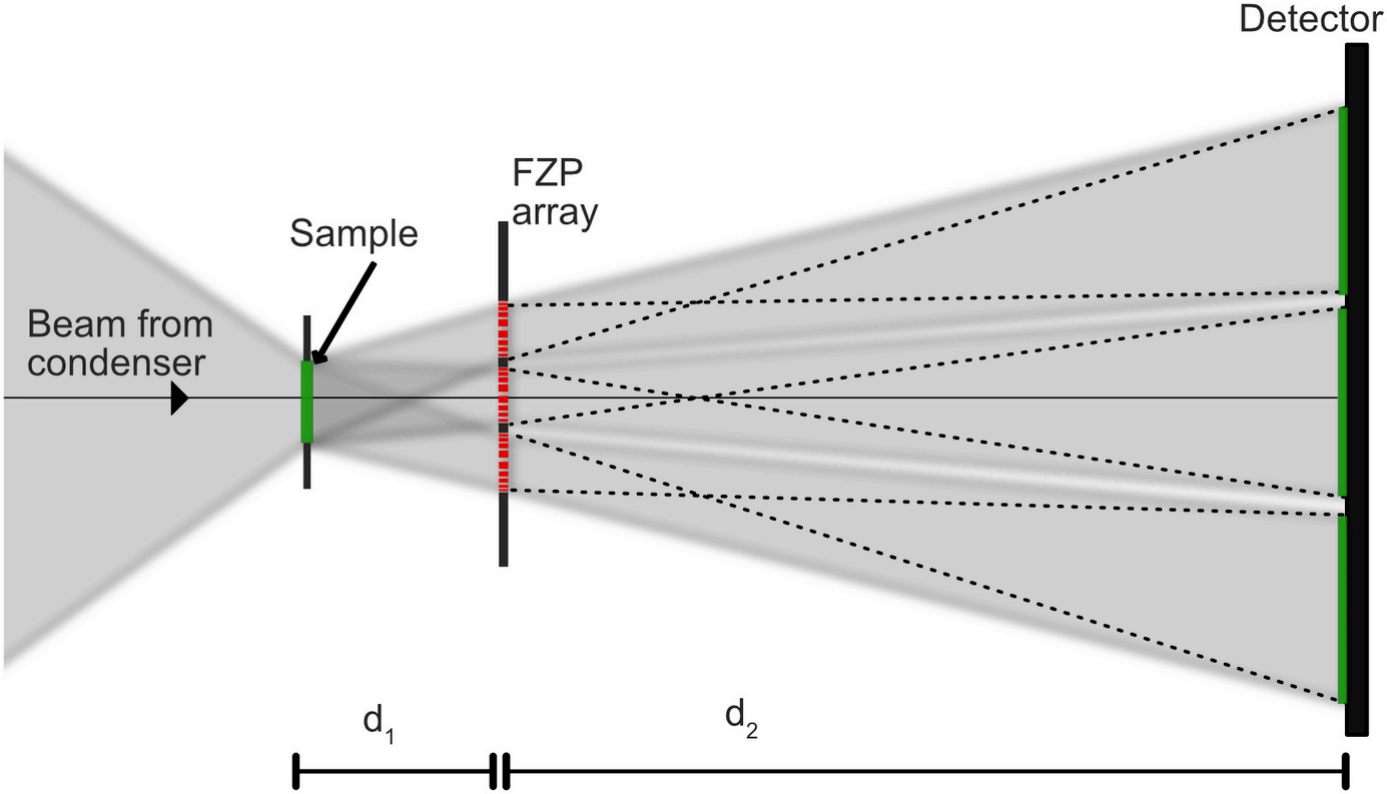
Illustration of the concept of a bank of objectives, where each objective is an FZP. The optical axes are parellel in the case shown.

A solution based on the fabrication of FZPs in a regular grid with a pitch corresponding to the diameter *D*, on a single substrate, is illustrated in Fig. 6 ▸. Such a design can be manufactured using existing lithography techniques. This approach provides a compact, easy-to-install solution, and can be readily integrated into most existing instruments. To prevent overlap between the images produced by individual FZPs, the FOV should be limited to the diameter of the FZP. With an NA of 0.002 (for Δ*r* = 100 nm) the number of individual FZPs required to match the full divergence is larger than for the corresponding CRL case, approaching 600. While this target is demanding, we believe that the technical feasibility is primarily determined by that of an isolated FZP.

It appears that the aberrations due to off-axis imaging will become worse for the outermost FZPs in the grid with the design shown in Fig. 6 ▸. As mentioned, we estimate that the aberration will still be tolerable, but this needs verification. To avoid off-axis aberrations, and ensure complete spatial and angular sampling, we consider fabrication of the bank of FZPs on a curved substrate with a radius of curvature of *d*_1_. Patterning an array of FZPs on a curved substrate has been demonstrated for visible-light applications (Moghimi *et al.*, 2015 ▸; Low *et al.*, 2022 ▸). One concern for neutrons is that the high-aspect-ratio nanostructures when tilted on a curved substrate at some point will start to violate the scalar diffraction approximation (Ali & Jacobsen, 2020 ▸).

In conclusion, for monochromatic neutron beam operation, this type of objective bank is well suited for inclusion in a micro-CT neutron microscope based on the Wolter-I condenser. The available space around the sample, as defined by the focal length, complies with the design range. It also matches the FOV and the magnification enables the combination of a micrometre-size spatial resolution with a magnification of five or more. In comparison with the CRL bank, the FZP solution on a flat surface is more elegant and technically easier to scale up. Simulations are required to learn if the off-axis aberrations are acceptable; if not, research and development is required in FZP technology on curved surfaces.

## 4. An objective bank for large energy bandwidth imaging

CRLs and FZPs are both chromatic-type optics. When changing the neutron energy the focal length and the magnification change. Superposing the signals from different energies leads to variations in focal length and in magnification known as longitudinal and lateral (also known as transverse) chromatic aberration, respectively. The former blur manifests itself in a constant blur across the FOV while the latter blur increases with the distance to the optical axis. In the thin-lens limit we have (with magnification unsigned) 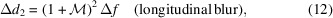

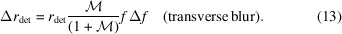
Here Δ*r*_det_ is the shift on the detector expressed in terms of the distance r_det_ to the optical axis. (The different functional dependence of focal length *f* on energy implies that the FZP exhibits only half the blur of the CRL.)

**Figure 7:**
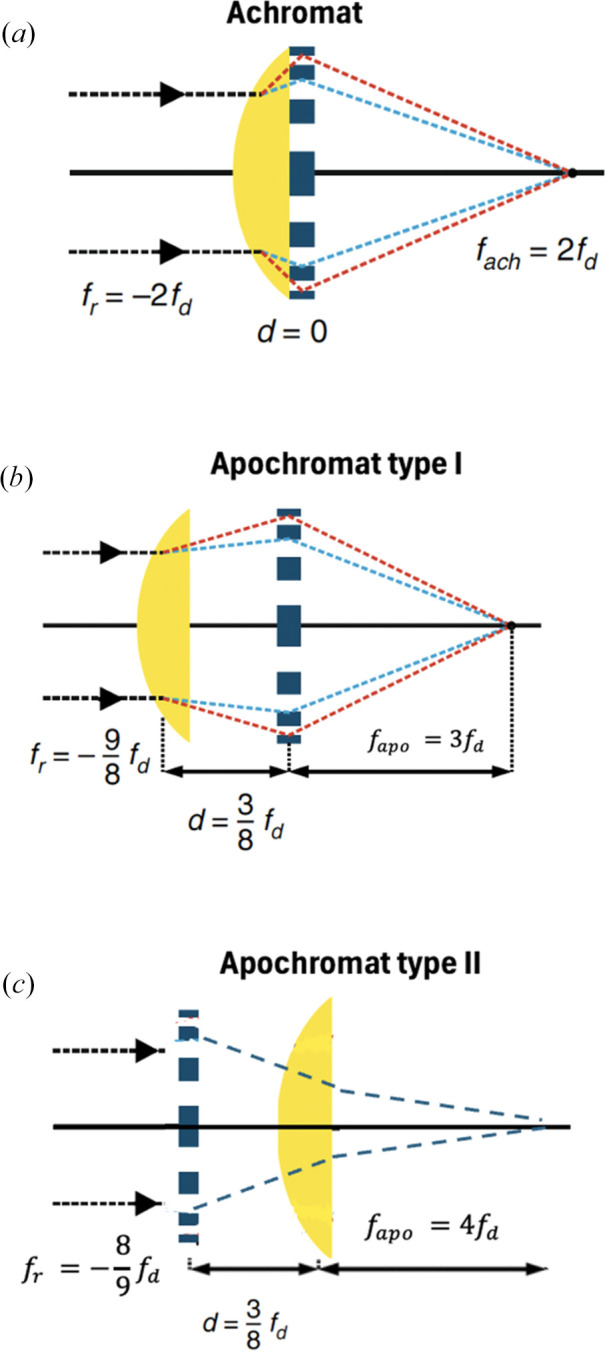
Sketch of the principle of a neutron achromat (*a*) and neutron apo­chromats of type I (*b*) and II (*c*) in a focusing geometry. Red and blue lines indicate rays with energies that are 10% different. Relationships between key optical distances are indicated (see main text). Adapted from Sanli *et al.* (2023 ▸) under a CC4 license (https://creativecommons.org/licenses/by/4.0/).

This chromatic aberration is clearly a critical issue for high-spatial-resolution imaging work, as it reduces the available flux by several orders of magnitude. In visible-light optics, a combination of optics with different dispersion powers is used to enable larger-bandwidth imaging. Similar optics have been introduced for X-rays: Skinner (2001 ▸) and Wang *et al.* (2003 ▸) proposed achromats comprising a positive diffractive lens adjacent to a negative refractive lens. In the thin-lens limit, the focal length of the combined optics (*f*_achr_) is independent of energy to second order provided that *f*_CRL_ = −2*f*_FZP_. The resulting focal length is then *f*_achr_ = 2*f*_FZP_. This is illustrated in Fig. 7 ▸(*a*). Likewise Skinner (2004 ▸) and Chapman & Bajt (2021 ▸) discuss apochromats, where the two optical elements are separated by a distance *d*. By suitable tuning the combined ‘focal length’, *f*_apo_ becomes independent of energy to third order.

The latter paper distinguishes two types of apochromats, referred to as type I and type II. In type I the refractive lens is upstream of the diffractive lens, illustrated in Fig. 7 ▸(*b*), and in type II the diffractive lens is followed by the refractive lens [Fig. 7 ▸(*c*)]. Key optical parameters, such as the distance from the exit of the combined optics to the focal point are summarized in the figure. Notably the entire optic becomes significantly longer than for the apochromat solutions and the FOV may also be impacted. The paper also extends the theory to thick lenses.

Type-I achromats and apochromats were both experimentally demonstrated with X-rays by Kubec *et al.* (2022 ▸) and Sanli *et al.* (2023 ▸). These optics were fabricated using electron beam lithography, electroplating and two-photon polymerization-induced lithography. High-resolution scanning transmission X-ray microscopy imaging has been demonstrated using both these lenses over a wide bandwidth range, demonstrating the suitability of this class of optics for broadband high-resolution imaging.

Poulsen *et al.* (2014 ▸) proposed to create broadband neutron optics in a similar way by combining a converging FZP and a diverging CRL. In this section we first summarize these results. Then we explore the concept of a bank of neutron achromats.

### 4.1. A neutron achromat or apochromat

Poulsen *et al.* (2014 ▸) determined the optical properties of an achromat and a type II apochromat using ray transfer matrix analysis. In that article, the CRL is approximated by two parameters: the thin-lens focal length and the thickness *NT*. By differentiating the combined focal length with respect to energy, *analytical expressions for the thick-lens case* are derived for the parameters for achromatic focusing (focal lengths, distance *detc.*).

Next this analysis is repeated for an imaging set-up. Notably, the parameters now depend on magnification. To illustrate this the relation between *f*_CRL_ and *f*_FZP_ for the achromat is 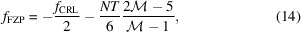
which in the thin-lens limit corresponds to the expression in Fig. 7 ▸(*a*).

**Figure 8:**
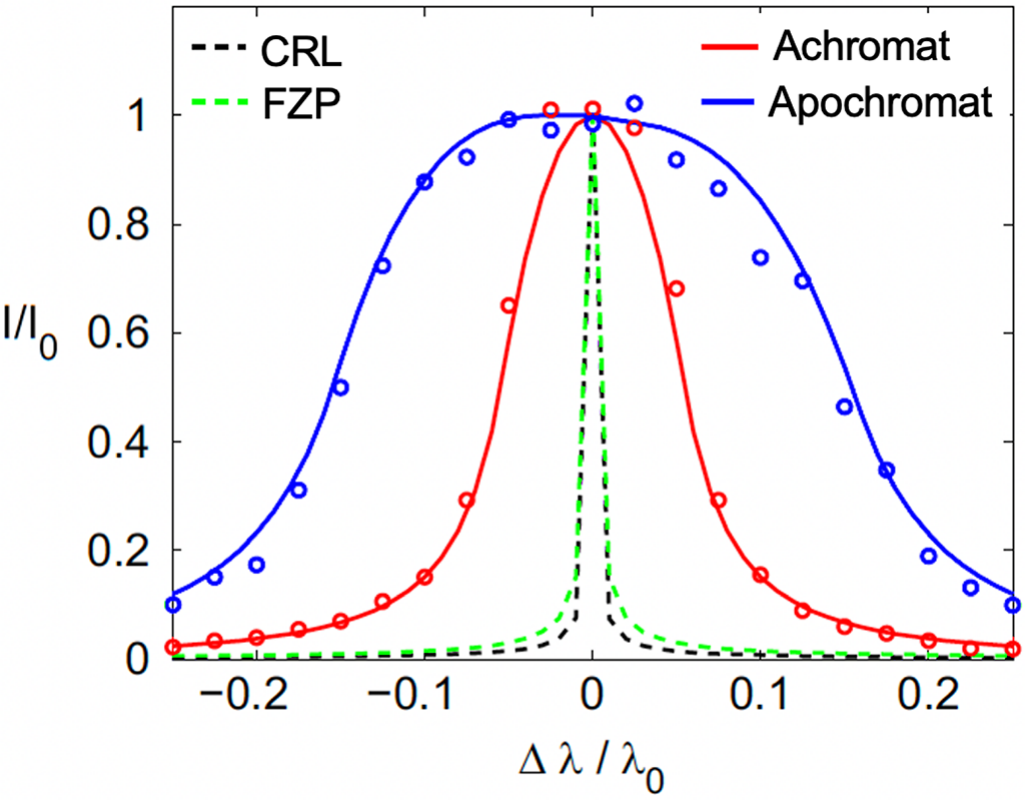
Chromatic aberration of a neutron achromat (red symbols) and a type II apochromat (blue). Shown is the variation in intensity in the image as a function of neutron wavelength. Full lines represent results from ray transfer matrix analysis, and circles are results from Fourier optics simulations. For comparison the dispersion of an FZP (dashed green line) and a CRL optic (dashed black line) are also shown. The data originate from Poulsen *et al.* (2014 ▸).

The paper proceeds with detailed simulations of a λ = 6 Å set-up with a fixed 5 mm-diameter FZP, a focal length of 1 m and a magnification of three. An example of the results is reproduced in Fig. 8 ▸. The energy bandwidth, as defined by the FWHM, is 12% and 31% in the two cases. This work also documented that the efficiencies of the achromat/apochromat were almost as good as the FZP on its own (37% efficiency for both achromat and apochromat). This entire work was further substantiated with Fourier optics simulations, validating the geometrical optics work.

In order to minimize the wavelength-dependent distortion due to gravitation, a neutron prism (wedge) could be used as both these effects grow proportionally to λ^2^ (see Hammouda & Mildner, 2007 ▸). For an implementation at a time-of-flight-based imaging instrument such as ODIN, the gravitational effects may also be corrected for during the data analysis.

### 4.2. A bank of neutron achromats or apochromats

Based on the results of Sections 3 and 4.1 we can now discuss the prospect of an objective comprising a bank of neutron achromats or apochromats.

 (i) *Spatial and angular coverage.* FZPs and CRLs can be made to match in terms of NA, FOV and focal lengths. The achromat and apochromat solutions can provide a full coverage of the angular range defined by the divergence of the condensed beam. The small angular gaps in the coverage between the individual units correspond to finite angular step sizes, which is not an issue for the reconstruction algorithm. The FOV of each unit will be governed by the CRL and will match the specified 4 × 4 mm. If possible, to avoid cross talk between units the incident beam should be confined to 4 × 4 mm by a slit.

 (ii) *Efficiency and energy band.* As mentioned the CRLs will effectively be transparent lenses, implying that the efficiency is governed by the zone plates. Theoretically the overall efficiency can be better than 30%. The theoretical bandwidths for achromats and apochromats are displayed in Fig. 8 ▸.

 (iii) *Spatial resolution.* The diffraction limit and known aberrations in the FZP are all below 2 µm. This specification is also easily achievable in terms of vibration control and temperature stability. Hence, the limitation on spatial resolution will be precision in manufacturing and assembly. Assuming a flat surface design is used, the key performance parameter for the FZP is the AR of the outermost zone (see Fig. 5 ▸). The divergent CRL is the inverse of the design shown in Fig. 4 ▸; it can be realized *e.g.* by free standing but identical parabolic-shaped balls made of diamond or MgF_2_. These balls are then organized in space by means of mechanical guides. The design is beyond the scope of this article, but we assume that a manufacturing accuracy of a few micrometres is within reach.

 (iv) *Detector technology.* The long focal lengths of the achromat/apochromats implies that the magnification will be below 10 at existing beamlines. A CMOS camera coupled to a scintillator screen is well adapted to this case in terms of pixel size. The neutron microscope will require a massive coverage by detectors, defined by say 400 simultaneous projections each aiming at resolving a 1000 × 1000 area. Adapting the focal lengths for the individual projections all data may be acquired on one large planar detector with say 1 gigapixels. X-ray imaging scintillator-based cameras with 150–600 megapixels and 1–4 µm pixel size are now in routine use (Kameshima & Hatsui, 2022 ▸; Gellert & *et al.*, 2025 ▸). A similar technological development within neutron imaging detectors can be expected in the coming years.

As an alternative, one may aim to increase the magnification such that single-neutron-counting detectors with larger pixel sizes become viable. This will improve the signal-to-noise ratio.

 (iv) *Choice of broadband optics.* The three alternative set-ups illustrated in Fig. 7 ▸ have different merits. The achromat solution is superior in relation to neutron magnification: see the discussion on detectors above. The apochromats provide a broader energy bandwidth.

## 5. Discussion

Full-scale simulation of the neutron transport and image formation in the microscope are needed for validating performance and for detailed optimization. This is beyond the scope of this article. We have conceptualized the method, listed the perceived key limitations and provided a discussion of the feasibility.

### 5.1. Condenser

The Wolter-I optic design considered here for the neutron condenser was optimized for telescopes, *i.e.* for an extremely low divergent beam. An elliptic–hyperbolic combination of Wolter optics would be better suited for divergent beams (Liu *et al.*, 2012 ▸). Moreover, specifically in relation to ODIN, it is obvious that a better match can be made between the exit of the neutron guide and the dimensions of the optic, *e.g.* by replacing the elliptical part by a straight section. The rationale for the choice of model system made in Section 2.1 is that all parameters are copied from an existing instrument. This gives confidence in the conclusion that a gain in neutron flux of up to a factor 100 is feasible.

We emphasize that the neutron condenser is relevant without the objective. When applied in front of a pinhole-camera-type instrument, it enables increasing the beam divergence homogeneously and thus achieving a larger FOV despite a relatively low incoming divergence, as found typically in neutron imaging instruments at the end of a neutron guide. It can be used as a means of efficient pinhole-to-pinhole beam transport, for efficient scanning-type mapping experiments, for high-resolution measurements of thin samples being basically in contact with the detector, for medium-resolution high-intensity imaging and for high-resolution neutron capture imaging.

### 5.2. Objective

In terms of alternatives to the proposed optics, we emphasize that reflective optics being achromatic are ideal. However, also for reflective optics a multiplexed objective design may be relevant to improve angular resolution. We note that a full-field X-ray microscope based on Kirkpatrick–Baez mirror optics has shown sub-micrometre resolution over a broad energy range (Matsuyama *et al.*, 2019 ▸). However, such solutions require the use of a thick substrate and we are not familiar with solutions that allow multiplexing such optics.

An alternative solution to overcoming the issue of achromaticity is to exploit the fact that neutrons have finite velocities and change configuration of the objective during the duration of the pulse. This is discussed by Poulsen *et al.* (2014 ▸).

Finally, we emphasize that the objective bank is relevant without the condenser and in fact may be used for SANS imaging with a highly collimated incident beam. In this case the specifications for NA can be relaxed in order to increase the *Q* range.

### 5.3. Data analysis

The high divergence of the beam on the sample implies that the depth of focus will often be smaller than the thickness of the sample. This implies that the classical cone-beam geometry ansatz in tomography does not apply. Moreover, some of the projections provided by the objective bank will be out-of-plane by up to 1.5°. Hence, tomographic reconstruction will require a new algorithm; this however is straightforward using a linear algebraic approach and an optimization based on the resemblance of experimental data and simulated data from a forward model of a digital sample (Hansen *et al.*, 2021 ▸). *A priori* information about the sample structure may be included, *e.g.* by adding a regularizing term.

The Crowther criterion states that to provide a 3D tomographic reconstruction of an object of diameter *D* with a spatial resolution of Δ*x*, the angular step size Δω during a 180° rotation must fulfill 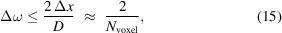
where 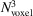 is the number of voxels in the reconstructed volume. In the achromat solution presented in Section 4, projections are acquired with an angular width given by the NA of the FZP. Notably 4 mrad corresponds to *N*_voxel_ = 500, which is well matched to many science cases. In contrast, if projections implied integration over the full divergence of 3°, *N*_voxel_ = 35, which is often too small. For this reason we argue that the multiplexed objective at times may be favorable even if it becomes technically feasible to make a nested mirror-type objective.

The fact that the beam is focused within the sample also opens up opportunities for other types of scans including depth scanning and local tomography.

## 6. Conclusion

The large divergence and large source size inherent to neutron sources has been a major limitation in the development of full-field neutron microscopy. In order to fulfill the simultaneous requirements for spatial resolution, working distance, efficiency and broad-band operation, we have presented a design involving a combination of a nested supermirror condenser and an objective bank comprising neutron achromats. We argue that this solution is valid in terms of the principles of optics and we have discussed challenges and solutions for implementation. For tomography a key advantage is that hundreds of projections are acquired simultaneously with an angular integration that matches reconstructions of 500^3^ voxels. The optics concepts are also valid for diffraction-based imaging.

## Figures and Tables

**Table 1:** Optical properties of materials for neutron CRLs ρ is density, δ the refractive index decrement and NA_max_ = (2δ)^1/2^ the maximum NA. μ is the linear attenuation coefficient for a single crystal that exhibits no diffraction. The numbers are calculated for a wavelength of 4 Å.

Material	ρ (g cm^−3^)	δ (10^−6^)	NA_max_ (10^−3^)	μ (m^−1^)
C (diamond)	3.51	29.3	10.8	0.08
MgF_2_	3.15	7.1	5.02	0.44

**Table 2:** Neutron optical parameters at a wavelength of 4 Å for materials suitable for manufacturing a neutron FZP The linear attenuation coefficients represent the powder case.

Material	δ (10^−6^)	μ (m^−1^)	Δ*T*_π_ (µm)
Nickel	24	260	8.34
Diamond	29.8	97.9	6.71
Silicon	5.28	13	37.8

**Table 3:** Geometry of primary and secondary conical shells in the condenser design visualized in Fig. 1 ▸

Conic	Length_1_ (mm)	*r*_1, in_ (mm)	*r*_1, out_ (mm)	Length_2_ (mm)	*r*_2, in_ (mm)	*r*_2, out_ (m)
1	459.2	12.1	10.9	459.2	10.9	7.1
2	405.3	13.6	12.3	405.2	12.3	8.6
3	362.6	15.0	13.8	362.6	13.8	10.0
4	328.1	16.5	15.2	328.1	15.2	11.5
5	299.6	17.9	16.7	299.6	16.7	12.9
6	275.7	19.4	18.1	275.7	18.1	14.4
7	255.3	20.8	19.6	255.2	19.6	15.8
8	237.7	22.3	21.0	237.7	21.0	17.3
9	222.4	23.7	22.5	222.3	22.5	18.7
10	208.9	25.2	23.9	208.9	23.9	20.2
11	197.0	26.6	25.4	196.9	25.4	21.6
12	186.3	28.1	26.8	186.3	26.8	23.1
13	176.8	29.5	28.3	176.7	28.3	24.5
14	168.2	31.0	29.7	168.1	29.7	26.0
15	160.4	32.4	31.2	160.3	31.2	27.4
16	153.2	33.9	32.6	153.2	32.6	28.9
17	146.7	35.3	34.1	146.7	34.1	30.3
18	140.7	36.8	35.5	140.7	35.5	31.8
19	135.2	38.2	37.0	135.2	37.0	33.2
20	130.1	39.7	38.4	130.1	38.4	34.7
21	125.4	41.1	39.9	125.4	39.9	36.1
22	121.0	42.6	41.3	121.0	41.3	37.6
23	116.9	44.0	42.8	116.9	42.8	39.0
24	113.1	45.5	44.2	113.0	44.2	40.5
25	109.5	46.9	45.7	109.4	45.7	41.9
26	106.1	48.4	47.1	106.1	47.1	43.4
27	103.0	49.8	48.6	102.9	48.6	44.8
28	100.0	51.2	50.0	99.9	50.0	46.3

## Appendix A Appendix A

The geometry of primary and secondary conical shells is presented in Table 3 ▸.

## References

[bb1] Abir, M., Hussey, D. S. & Khaykovich, B. (2020). *J. Imaging* **6**, 100. 10.3390/jimaging6100100 PMC8321192 34460541

[bb2] Ali, S. & Jacobsen, C. (2020). *J. Opt. Soc. Am. A* **37**, 374–383. 10.1364/JOSAA.380925 PMC7080321 32118920

[bb3] Allman, B., McMahon, P., Nugent, K., Paganin, D., Jacobson, D., Arif, M. & Werner, S. A. (2000). *Nature* **408**, 158–159. 10.1038/35041626 11089960

[bb4] Altissimo, M. (2004). *Microelectron. Eng.* **73–74**, 644–650.

[bb5] Andersen, K. H., Argyriou, D. N., Jackson, A. J., Houston, J., Henry, P. F., Deen, P. P., Toft-Petersen, R., Beran, P., Strobl, M., Arnold, T., Wacklin-Knecht, H., Tsapatsaris, N., Oksanen, E., Woracek, R., Schweika, W., Mannix, D., Hiess, A., Kennedy, S., Kirstein, O., Petersson Årsköld, S., Taylor, J., Hagen, M. E., Laszlo, G., Kanaki, K., Piscitelli, F., Khaplanov, A., Stefanescu, I., Kittelmann, Th., Pfeiffer, D., Hall-Wilton, R., Lopez, C. I., Aprigliano, G., Whitelegg, L., Moreira, F. Y., Olsson, M., Bordallo, H. N., Martín-Rodríguez, D., Schneider, H., Sharp, M., Hartl, M., Nagy, G., Ansell, S., Pullen, S., Vickery, A., Fedrigo, A., Mezei, F., Arai, M., Heenan, R. K., Halcrow, W., Turner, D., Raspino, D., Orszulik, A., Cooper, J., Webb, N., Galsworthy, P., Nightingale, J., Langridge, S., Elmer, J., Frielinghaus, H., Hanslik, R., Gussen, A., Jaksch, S., Engels, R., Kozielewski, T., Butterweck, S., Feygenson, M., Harbott, P., Poqué, A., Schwaab, A., Lieutenant, K., Violini, N., Voigt, J., Brückel, T., Koenen, M., Kämmerling, H., Babcock, E., Salhi, Z., Wischnewski, A., Heynen, A., Désert, S., Jestin, J., Porcher, F., FabrèGes, X., FabrèGes, G., Annighöfer, B., Klimko, S., Dupont, Th., Robillard, Th., Goukassov, A., Longeville, S., Alba-Simionesco, Ch., Bourges, P., Guyon Le Bouffy, J., Lavie, P., Rodrigues, S., Calzada, E., Lerche, M., Schillinger, B., Schmakat, P., Schulz, M., Seifert, M., Lohstroh, W., Petry, W., Neuhaus, J., Loaiza, L., Tartaglione, A., Glavic, A., Schütz, S., Stahn, J., Lehmann, E., Morgano, M., Schefer, J., Filges, U., Klauser, Ch., Niedermayer, Ch., Fenske, J., Nowak, G., Rouijaa, M., Siemers, D. J., Kiehn, R., Müller, M., Carlsen, H., Udby, L., Lefmann, K., Birk, J. O., Holm-Dahlin, S., Bertelsen, M., Hansen, U. B., Olsen, M. A., Christensen, M., Iversen, K., Christensen, N. B., Rønnow, H. M., Freeman, P. G., Hauback, B. C., Kolevatov, R., Llamas-Jansa, I., Orecchini, A., Sacchetti, F., Petrillo, C., Paciaroni, A., Tozzi, P., Zanatta, M., Luna, P., Herranz, I., del Moral, O. G., Huerta, M., Magán, M., Mosconi, M., Abad, E., Aguilar, J., Stepanyan, S., Bakedano, G., Vivanco, R., Bustinduy, I., Sordo, F., Martínez, J. L., Lechner, R. E., Villacorta, F. J., Šaroun, J., Lukáš, P., Markó, M., Zanetti, M., Bellissima, S., del Rosso, L., Masi, F., Bovo, C., Chowdhury, M., De Bonis, A., Di Fresco, L., Scatigno, C., Parker, S. F., Fernandez-Alonso, F., Colognesi, D., Senesi, R., Andreani, C., Gorini, G., Scionti, G. & Schreyer, A. (2020). *Nucl. Instrum. Methods Phys. Res. A* **957**, 163402.

[bb7] Antipov, S., Baryshev, S. V., Butler, J. E., Antipova, O., Liu, Z. & Stoupin, S. (2016). *J. Synchrotron Rad.* **23**, 163–168. 10.1107/S1600577515020639 PMC4733930 26698059

[bb8] Bacak, M., Valsecchi, J., Čapek, J., Polatidis, E., Kaestner, A., Arabi-Hashemi, A., Kruk, I., Leinenbach, C., Long, A., Tremsin, A., Vogel, S., Watkins, E. & Strobl, M. (2020). *Mater. Des.* **195**, 109009.

[bb9] Beguiristain, H. R., Anderson, I. S., Dewhurst, C. D., Piestrup, M. A., Cremer, J. T. & Pantell, R. H. (2002). *Appl. Phys. Lett.* **81**, 4290–4292.

[bb10] Cederström, B., Ribbing, C. & Lundqvist, M. (2005). *J. Synchrotron Rad.* **12**, 340–344. 10.1107/S0909049504034181 15840919

[bb11] Cereser, A., Strobl, M., Hall, S. A., Steuwer, A., Kiyanagi, R., Tremsin, A. S., Knudsen, E. B., Shinohara, T., Willendrup, P. K., da Silva Fanta, A. B., Iyengar, S., Larsen, P. M., Hanashima, T., Moyoshi, T., Kadletz, P. M., Krooß, P., Niendorf, T., Sales, M., Schmahl, W. W. & Schmidt, S. (2017). *Sci. Rep.* **7**, 9561. 10.1038/s41598-017-09717-w PMC5572055 28842660

[bb12] Chang, C. & Sakdinawat, A. (2014). *Nat. Commun.* **5**, 4243. 10.1038/ncomms5243 24970569

[bb13] Chapman, H. N. & Bajt, S. (2021). *Proc. R. Soc. London A.* **477**, 20210334. 10.1098/rspa.2021.0334 PMC8277474 34276244

[bb14] Christensen, F. E., Jakobsen, A. C., Brejnholt, N., Madsen, K. K., Hornstrup, A., Westergaard, N. J. S., Momberg, J., Koglin, J., Fabricant, A. M., Stern, M., Craig, W. W., Pivovaroff, M. J. & Windt, D. (2011). *Proc. SPIE* **8147**, 81470U.

[bb15] Craig, W. W., An, H., Blaedel, K. L., Christensen, F. E., Decker, T. A., Fabricant, A., Gum, J., Hailey, C. J., Hale, L., Jensen, C. B., Koglin, J. E., Mori, K., Nynka, M., Pivovaroff, M. J., Sharpe, M. V., Stern, M., Tajiri, G. & Zhang, W. W. (2011). *Proc. SPIE* **8147**, 81470H.

[bb16] Cremer, J. T., Piestrup, M. A., Park, H., Gary, C. K., Pantell, R. H., Glinka, C. J. & Barker, J. G. (2005). *Appl. Phys. Lett.* **87**, 161913.

[bb6] De Andrade, V., Nikitin, V., Wojcik, M., Deriy, A., Bean, S., Shu, D., Mooney, T., Peterson, K., Kc, P., Li, K., Ali, S., Fezzaa, K., Gürsoy, D., Arico, C., Ouendi, S., Troadec, D., Simon, P., De Carlo, F. & Lethien, C. (2021). *Adv. Mater.* **33**, 2008653. 10.1002/adma.202008653 33871108

[bb71] Dhanalakshmi Veeraraj, M. R., Qu, D., Zhao, S., Qi, P., Jefimovs, K., Busi, M., Kohlbrecher, J., David, C., Strobl, M. & Vila-Comamala, J. (2025). *Sci. Rep.* **15**, 8408. 10.1038/s41598-025-92329-6 PMC11897255 40069214

[bb18] Di Fabrizio, E., Romanato, F., Gentili, M., Cabrini, S., Kaulich, B., Susini, J. & Barrett, R. (1999). *Nature* **401**, 895–898.

[bb17] Eskildsen, M. R., Gammel, P. L., Isaacs, E. D., Detlefs, C., Mortensen, K. & Bishop, D. J. (1998). *Nature* **391**, 563–566.

[bb19] Gellert, N. C., Kantor, I., Christensen, T. E. K., Rodriguez-Palomo, A., Thomson, E. L., Høeg, A. L., Niese, S., Olsen, U. L., Dahl, A. B., Dyrby, T. B., Birkedal, H., Poulsen, H. F. & Mokso, R. (2025). *Opt. Express* **33**, 42221–42239. 10.1364/OE.565345 41215069

[bb20] Hammouda, B. & Mildner, D. F. R. (2007). *J. Appl. Cryst.* **40**, 250–259.

[bb21] Hansen, P. C., Jørgensen, J. S. & Lionheart, W. R. B. (2021). Editors. *Computed Tomography: Algorithms, Insight, and Just Enough Theory.* SIAM.

[bb22] Harrison, F., Craig, W., Christensen, F., Hailey, C., Zhang, W., Boggs, S., Stern, D., Cook, W., Forster, K., Giommi, P., Grefenstette, B., Kim, Y., Kitaguchi, T., Koglin, J., Madsen, K., Mao, P., Miyasaka, H., Mori, K., Perri, M., Pivovaroff, M., Puccetti, S., Rana, V., Westergaard, N., Willis, J., Zoglauer, A., An, H., Bachetti, M., Barrière, N., Bellm, E., Bhalerao, V., Brejnholt, N., Fuerst, F., Liebe, C., Markwardt, C., Nynka, M., Vogel, J., Walton, D., Wik, D., Alexander, D., Cominsky, L., Hornschemeier, A., Hornstrup, A., Kaspi, V., Madejski, G., Matt, G., Molendi, S., Smith, D., Tomsick, J., Ajello, M., Ballantyne, D., Baloković, M., Barret, D., Bauer, F., Blandford, R., Brandt, W., Brenneman, L., Chiang, J., Chakrabarty, D., Chenevez, J., Comastri, A., Dufour, F., Elvis, M., Fabian, A., Farrah, D., Fryer, C., Gotthelf, E., Grindlay, J., Helfand, D., Krivonos, R., Meier, D., Miller, J., Natalucci, L., Ogle, P., Ofek, E., Ptak, A., Reynolds, S., Rigby, J., Tagliaferri, G., Thorsett, S., Treister, E. & Urry, C. (2013). *ApJ* **770**, 103.

[bb23] Hiroi, K., Shinohara, T., Hayashida, H., Parker, J., Su, Y., Oikawa, K., Kai, T. & Kiyanagi, Y. (2018). *Physica B* **551**, 146–151.

[bb24] Howells, M., Jacobsen, C., Warwick, T. & Van den Bos, A. (2017). *Science of Microscopy*, edited by P. Hawkes & J. Spence, pp. 835–926. Springer.

[bb25] Hussey, D., Abir, M., Cook, J., Jacobson, D., LaManna, J. M., Kilaru, K., Ramsey, B. D. & Khaykovich, B. (2021). *Nucl. Instrum. Methods Phys. Res. A* **987**, 164813. 10.1016/j.nima.2020.164813 PMC8634521 34866719

[bb26] Jacobsen, H., Lieutenant, K., Zendler, C. & Lefmann, K. (2013). *Nucl. Instrum. Methods Phys. Res. A* **717**, 69–76.

[bb27] Jorba, P., Schulz, M., Hussey, D., Abir, M., Seifert, M., Tsurkan, V., Loidl, A., Pfleiderer, C. & Khaykovich, B. (2019). *J. Magn. Magn. Mater.* **475**, 176–183. 10.1016/j.jmmm.2018.11.086 PMC8751646 35023885

[bb28] Kallmann, H. (1948). *Research* **1**, 254–260. 18910022

[bb29] Kameshima, T. & Hatsui, T. (2022). *J. Phys. Conf. Ser.* **2380**, 012094.

[bb30] Kardjilov, N., Manke, I., Strobl, M., Hilger, A., Treimer, W., Meissner, M., Krist, T. & Banhart, J. (2008). *Nat. Phys.* **4**, 399–403.

[bb31] Kardjilov, N., Manke, I., Woracek, R., Hilger, A. & Banhart, J. (2018). *Mater. Today* **21**, 652–672.

[bb32] Karimi, V., Qvistgaard, C., Schmidt, S., Wolfertz, A., Parker, J. D., Tetsuya, K., Hayashida, H., Shinohara, T., De Angelis, S., Tengattini, A., Sharma, R., Fedrigo, A., Helfen, L., Morgen, P., Andersen, S. M. & Theil Kuhn, L. (2025). *Appl. Mater. Interfaces* **17**, 50742–50752. 10.1021/acsami.5c11706 40886136

[bb33] Kearney, P. D., Klein, A. G., Opat, G. I. & Gähler, R. (1980). *Nature* **287**, 313–314.

[bb34] Khaykovich, B., Gubarev, M., Bagdasarova, Y., Ramsey, B. & Moncton, D. (2011). *Nucl. Instrum. Methods Phys. Res. A* **631**, 98–104.

[bb35] Knudsen, E., Della Monica Ferreira, D., Westergaard, N., Massahi, S., Christensen, F., Ferreira, I., Shortt, B. & Spiga, D. (2018). *Proc. SPIE* **10699**, 106993S.

[bb36] Kubec, A., Zdora, M. C., Sanli, U. T., Diaz, A., Vila-Comamala, J. & David, C. (2022). *Nat. Commun.* **13**, 2–8. 10.1038/s41467-022-28902-8 PMC8921332 35288546

[bb37] Larsen, C. B., Samothrakitis, S., Woracek, R., Polatidis, E., Čapek, J., Upadhyay, M. V., Tovar, M., Schmidt, S. & Strobl, M. (2025). *Acta Mater.* **289**, 120869.

[bb38] Leemreize, H., Knudsen, E. B., Birk, J. O., Strobl, M., Detlefs, C. & Poulsen, H. F. (2019). *J. Appl. Cryst.* **52**, 1299–1311.

[bb39] Lehmann, E. H. (2015). *Neutron News* **26**(2), 2. 10.1080/10448632.2015.1028273 PMC11015479 38617044

[bb40] Lehmann, E. H., Peetermans, S., Josic, L., Leber, H. & van Swygenhoven, H. (2014). *Nucl. Instrum. Methods Phys. Res. A* **735**, 102–109.

[bb41] Liu, D., Gubarev, M., Resta, G., Ramsey, B., Moncton, D. & Khaykovich, B. (2012). *Nucl. Instrum. Methods Phys. Res. A* **686**, 145–150.

[bb42] Liu, D., Hussey, D., Gubarev, M. V., Ramsey, B. D., Jacobson, D., Arif, M., Moncton, D. E. & Khaykovich, B. (2013). *Appl. Phys. Lett.* **102**, 183508.

[bb43] Low, M. J., Rohith, T. M., Kim, B., Kim, S.-W., Suchand Sandeep, C. S., Murukeshan, V. M. & Kim, Y.-J. (2022). *J. Opt.* **24**, 055401.

[bb44] Manke, I., Kardjilov, N., Schäfer, R., Hilger, A., Strobl, M., Dawson, M., Grünzweig, C., Behr, G., Hentschel, M., David, C., Kupsch, A., Lange, A. & Banhart, J. (2010). *Nat. Commun.* **1**, 125. 10.1038/ncomms1125 21119638

[bb45] Matsuyama, S., Yamada, J., Kohmura, Y., Yabashi, M., Ishikawa, T. & Yamauchi, K. (2019). *Opt. Express* **27**, 18318–18328. 10.1364/OE.27.018318 31252777

[bb46] Mildner, D. & Gubarev, M. (2011). *Nucl. Instrum. Methods Phys. Res. A* **634**, S7–S11.

[bb47] Moghimi, M. J., Fernandes, J., Kanhere, A. & Jiang, H. (2015). *Sci. Rep.* **5**, 15861. 10.1038/srep15861 PMC4626810 26515117

[bb48] Mohacsi, I., Vartiainen, I., Guizar-Sicairos, M., Karvinen, P., Guzenko, V. A., Müller, E., Kewish, C. M., Somogyi, A. & David, C. (2016). *Opt. Lett.* **41**, 281–284. 10.1364/OL.41.000281 26766694

[bb49] Opolka, A., Müller, D., Fella, C., Balles, A., Mohr, J. & Last, A. (2021). *Appl. Sci.* **11**, 7234.

[bb75] Østergaard, M., Naver, E. B., Kaestner, A., Willendrup, P. K., Brüel, A., Sørensen, H. O., Thomsen, J. S., Schmidt, S., Poulsen, H. F., Theil Kuhn, L. & Birkedal, H. (2023). *J. Appl. Cryst.* **56**, 673–682. 10.1107/S1600576723003011 PMC10241042 37284268

[bb50] Paganin, D. M., Sales, M., Kadletz, P. M., Kockelmann, W., Beltran, M. A., Poulsen, H. F. & Schmidt, S. (2023). *Phys. Rev. Appl.* **19**, 034005.

[bb51] Peetermans, S., King, A., Ludwig, W., Reischig, P. & Lehmann, E. H. (2014). *Analyst* **139**, 5765–5771. 10.1039/c4an01490a 25274183

[bb52] Pfeiffer, F., Grünzweig, C., Bunk, O., Frei, G., Lehmann, E. & David, C. (2006). *Phys. Rev. Lett.* **96**, 215505. 10.1103/PhysRevLett.96.215505 16803249

[bb53] Poulsen, S. O., Poulsen, H. F. & Bentley, P. M. (2014). *Nucl. Instrum. Methods Phys. Res. A* **767**, 415–420.

[bb54] Sacchetti, F., Altissimo, M., Petrillo, C., Di Fabrizio, E., Colleoni, S. & Ott, F. (2004). *Physica B* **350**, E447–E450.

[bb55] Sales, M., Shinohara, T., Sørensen, M. K., Knudsen, E. B., Tremsin, A., Strobl, M. & Schmidt, S. (2019). *J. Phys. D Appl. Phys.* **52**, 205001.

[bb56] Sales, M., Strobl, M., Shinohara, T., Tremsin, A., Kuhn, L. T., Lionheart, W. R. B., Desai, N. M., Dahl, A. B. & Schmidt, S. (2018). *Sci. Rep.* **8**, 2214. 10.1038/s41598-018-20461-7 PMC5797168 29396502

[bb57] Sanli, U. T., Rodgers, G., Zdora, M.-C., Qi, P., Garrevoet, J., Falch, K. V., Müller, B., David, C. & Vila-Comamala, J. (2023). *Light Sci. Appl.* **12**, 107. 10.1038/s41377-023-01157-8 PMC10160054 37142565

[bb58] Santisteban, J. R., Edwards, L., Steuwer, A. & Withers, P. J. (2001). *J. Appl. Cryst.* **34**, 289–297.

[bb59] Schmakat, P., Seifert, M., Schulz, M., Tartaglione, A., Lerche, M., Morgano, M., Böni, P. & Strobl, M. (2020). *Nucl. Instrum. Methods Phys. Res. A* **979**, 164467.

[bb60] Schroer, C. G. & Lengeler, B. (2005). *Phys. Rev. Lett.* **94**, 054802. 10.1103/PhysRevLett.94.054802 15783651

[bb61] Shen, J., Busi, M., Rauscher, P., Valsecchi, J. G. N., Nemeth, G. & Strobl, M. (2025). *Sci. Rep.* **15**, 18438. 10.1038/s41598-025-02806-1 PMC12106693 40419635

[bb62] Simons, H., Ahl, S. R., Poulsen, H. F. & Detlefs, C. (2017). *J. Synchrotron Rad.* **24**, 392–401. 10.1107/S160057751602049X PMC5330290 28244432

[bb63] Skinner, G. K. (2001). *Astron. Astrophys.* **375**, 691–700.

[bb64] Skinner, G. K. (2004). *Appl. Opt.* **43**, 4845–4853. 10.1364/ao.43.004845 15449471

[bb65] Strobl, M. (2015). *Phys. Procedia* **69**, 18–26.

[bb66] Strobl, M., Grünzweig, C., Hilger, A., Manke, I., Kardjilov, N., David, C. & Pfeiffer, F. (2008). *Phys. Rev. Lett.* **101**, 123902. 10.1103/PhysRevLett.101.123902 18851372

[bb67] Strobl, M., Heimonen, H., Schmidt, S., Sales, M., Kardjilov, N., Hilger, A., Manke, I., Shinohara, T. & Valsecchi, J. (2019). *J. Phys. D Appl. Phys.* **52**, 123001.

[bb69] Strobl, M. & Lehmann, E. (2024). Editors. *Neutron Imaging.* IOP Publishing.

[bb68] Strobl, M., Manke, I., Kardjilov, N., Hilger, A., Dawson, M. & Banhart, J. (2009). *J. Phys. D Appl. Phys.* **42**, 243001.

[bb70] Treimer, W. (2019). *Handbook of Advanced Nondestructive Evaluation.* Springer.

[bb72] Wang, Y., Yun, W. & Jacobsen, C. (2003). *Nature* **424**, 50–53. 10.1038/nature01756 12840754

[bb73] Willendrup, P. K. & Lefmann, K. (2020). *J. Neutron Res.* **22**, 1–16.

[bb74] Willendrup, P. K. & Lefmann, K. (2021). *J. Neutron Res.* **23**, 7–27.

